# Diagnostic variability of mental disorders in adolescence: A longitudinal study across transition boundary age

**DOI:** 10.1002/jcv2.70174

**Published:** 2026-10-09

**Authors:** Marta Magno, Donato Martella, Silvia Leone, Adriana Pastore, Cosmo Strozza, Elisa Caselani, Samuele Cortese, Miriam D’Addazio, Gwen Dieleman, Tomislav Franic, Suzanne Gerritsen, Athanasios Maras, Fiona McNicholas, Diane Purper‐Ouakil, Paramala Santosh, Ulrike M. E. Schulze, Cathy Street, Swaran Singh, Sabine Tremmery, Helena Tuomainen, Larissa S. van Bodegom, Dieter Wolke, Giovanni de Girolamo, the MILESTONE Consortium

**Affiliations:** ^1^ Unit of Epidemiological Psychiatry and Digital Mental Health IRCCS Istituto Fatebenefratelli Brescia Italy; ^2^ Department of Statistics and Quantitative Methods University of Milano‐Bicocca Milano Italy; ^3^ Unit of Adolescent Psychiatric Emergency Policlinico Hospital Bari Italy; ^4^ Interdisciplinary Centre on Population Dynamics University of Southern Denmark Odense Denmark; ^5^ Faculty of Medicine University of Southampton Southampton UK; ^6^ Erasmus Medical Center Rotterdam the Netherlands; ^7^ Department of Psychiatry Medical School University of Split Split Croatia; ^8^ ARQ National Psychotrauma Centre Diemen the Netherlands; ^9^ Lucena Clinic Saint John of God Dublin Ireland; ^10^ University College Dublin Belfield Dublin Ireland; ^11^ Children’s Health Ireland at Crumlin Dublin Ireland; ^12^ CHU Montpellier Saint Eloi and Colombière Hospitals Montpellier France; ^13^ INSERM U 1018 Centre for Epidemiology and Population Health (CESP) Villejuif France; ^14^ King’s College London London UK; ^15^ South London and Maudsley NHS Foundation Trust London UK; ^16^ HealthTracker Ltd Kent UK; ^17^ University of Ulm Ulm Germany; ^18^ Warwick Medical School University of Warwick Coventry UK; ^19^ Department of Neurosciences KU Leuven Leuven Belgium; ^20^ Department of Psychology University of Warwick Coventry UK

**Keywords:** adolescents, CAMHS, diagnosis, mental health, transiction

## Abstract

**Background:**

Correct psychiatric diagnoses represent a prerequisite for the efficacy of treatment, especially during adolescence, a critical period for the onset of mental health disorders. Diagnoses can evolve over time, influenced by factors such as changes in symptoms, treatment effects, and comorbidities. The transition from Child and Adolescent Mental Health Services (CAMHS) to Adult Mental Health Services (AMHS) presents challenges due to differences in diagnostic criteria and treatment approaches. This study explores whether and how psychiatric diagnoses change during this transition, aiming to examine the prevalence and composition of primary diagnoses and identify predictors of diagnostic change.

**Methods:**

From the MILESTONE project total sample, 770 participants aged 17–19 with available diagnoses and clinical data were included and assessed at four‐time points. We categorised participants into 10 diagnostic groups based on shared clinical features. Statistical analysis included multilevel logistic regression to examine diagnosis changes, with a Sankey plot to visualise transitions.

**Results:**

Clinically, anxiety/trauma disorders (25.1%) were the most common at baseline, followed by depression/bipolar (21.3%) and attention deficit hyperactivity disorder (15.6%). Most diagnoses remained stable over time, with showing high stability; depression/bipolar showed moderate stability (80.7%), while schizophrenia spectrum disorders and substance use disorders exhibited nearly perfect consistency (100% and 85.7%, respectively). Multilevel logistic regression revealed that higher externalising scores (OR = 2.90; *p* = 0.045) was a predictor of diagnostic change, while being assessed by a psychiatrist (*p* = 0.037) was an index of diagnostic stability over time.

**Conclusion:**

This study highlights the diagnostic stability of most psychiatric conditions during the transition from CAMHS to AMHS. Given the complexity of adolescent mental health, accurate and continuous assessment is crucial. Understanding the evolving nature of psychiatric symptoms during adolescence will improve diagnostic accuracy and inform better interventions, fostering long‐term mental health outcomes.

## Key Points

**What's known?**

- Psychiatric diagnoses often change over time due to limited or unreliable information, treatment effects, coexisting conditions, and evolving symptoms. The transition from adolescence to adulthood poses additional challenges, as diagnostic approaches differ between developmental stages.

**What's new?**

- Most diagnoses remained stable over time. Depression/bipolar showed moderate stability, while schizophrenia spectrum and substance use disorders exhibited nearly perfect consistency. Higher externalizing scores predicted diagnostic change, whereas being assessed by a psychiatrist indicated diagnostic stability.

**What's relevant?**

- Diagnostic stability during the transition from Children and Adolescent Mental Health Services to Adult Mental Health Services was notable. However, given the complexity of adolescent mental health, accurate and continuous assessment is essential to monitor disorder progression and enable timely intervention.

## INTRODUCTION

Establishing reliable psychiatric diagnoses with both cross‐sectional and longitudinal coherence is crucial (Robins & Guze, 1970). Current diagnostic systems, such as the Diagnostic and Statistical Manual of Mental Disorders (DSM‐5) and the International Classification of Diseases (ICD‐10), emphasise both standardised criteria and a longitudinal perspective (American Psychiatric Association, DSM‐5 Task Force, 2013; Gore et al., 2011; World, 2004). This approach is particularly important for disorders emerging in adolescence, a critical period for mental health. However, psychiatric diagnoses often change over time due to factors such as limited or unreliable information, treatment effects, coexisting conditions, and evolving symptomatology (Kendler, 1990; Kendler & Walsh, 1998; Kramer et al., 2008; McClellan & McCurry, 1999; Peralta & Cuesta, 2003). The transitions from adolescence to adulthood present additional challenges, as diagnoses differ between developmental stages. Some disorders may emerge, subside, or shift in presentation during this period, making continuity of diagnosis more complex. Prospective studies indicate that many adult psychiatric disorders originate in adolescence, with diagnoses either remaining stable or transitioning to new disorders (Copeland et al., 2009). Stable diagnoses suggest persistent disease processes, whereas transitions highlight reciprocal influences, such as anxiety and depression, or progression from childhood behavioural issues to adult mental disorders (Costello et al., 2003; Ferdinand et al., 1995; Goodwin et al., 2004; Kim‐Cohen et al., 2003; Lewinsohn et al., 1999; Sourander et al., 2005). Copeland and colleagues (Copeland et al., 2013), analysing longitudinal data from three prominent cohort studies (Costello et al., 2016; Fergusson et al., 1989; Poulton et al., 2015), found that early‐onset disorders increase the risk of later‐life psychiatric conditions threefold, based on samples ranging from childhood to early adulthood. While stable transitions were common, particularly for neurodevelopmental conditions such as attention‐deficit/hyperactivity disorder (ADHD), which showed greater diagnostic stability, psychopathological disorders displayed weaker continuity. Depression and anxiety frequently influence each other but contrary to past findings, behavioural disorders were not found to reliably predict adult mental disorders, and adolescent emotional symptoms rarely led to young adult substance disorders (Copeland et al., 2009).

This diagnostic fluidity and the accompanying shifts in treatment and care needs are particularly evident during the transition from Children and Adolescent Mental Health Services (CAMHS) to Adult Mental Health Services (AMHS). This transition often represents a significant shift in mental health care, as adult services typically involve different diagnostic criteria, treatment methodologies, and care expectations. For individuals with ADHD, these changes in diagnosis, treatment approaches, and support systems make the transition particularly challenging. Research on ADHD transitions highlights significant difficulties in maintaining continuity of care (Tatlow‐Golden et al., 2018). A case note review conducted in Ireland revealed inconsistencies in diagnoses and fragmented treatment, resulting in disruption of patient care (Tatlow‐Golden et al., 2018). Similarly, McNicholas et al. (2016) conducted a global systematic review on ADHD service transitions, emphasising the lack of standardised transition processes, which contributes to a lack of clarity and consistency in treatment (McNicholas et al., 2016). Singh et al. (2010) further examined these issues in a multi‐perspective study, identifying critical barriers such as poor coordination between child and adult services, as well as inadequate support during the transition period (Singh et al., 2010). These barriers have been found to impact patient outcomes negatively, particularly in terms of ongoing symptom management and overall well‐being (Gerritsen et al., 2022).

Despite these findings, a significant gap remains in the literature regarding whether patients experience actual diagnostic changes as they transition between services. To date, no study has systematically examined whether diagnostic labels shift over time due to differing criteria, clinician perspectives, or evolving symptom presentations. To examine and address the treatment gaps that arise during transitions, we conducted a longitudinal cohort study, referred as ‘Managing the Link and Strengthening Transition from Child to Adult Mental Healthcare’ project—the MILESTONE study (Signorini et al., 2017; Singh et al., 2017). The current study, however, presents new findings based on data collected as part of this broader initiative, which aimed to enhance the understanding and improve the processes of transitional care from CAMHS to AMHS across European healthcare systems. Our primary objective is to examine clinician‐diagnosed conditions based on clinical observations and medical records. We hypothesise that the prevalence and composition of primary psychiatric diagnoses will shift as patients transition from CAMHS to AMHS, driven by differences in diagnostic criteria, clinician perspectives, and the natural evolution of symptomatology. Specifically, we expect a significant proportion of individuals will receive a different diagnosis at follow‐up (T4): after 24 months of monitoring from the start of the study compared to intake. Prior literature on diagnostic instability in youth mental health points to several domains likely to influence whether a diagnosis remains stable over time. Patient sex has been linked to differential symptom expression and diagnostic bias, with girls more likely to present with predominantly inattentive, internalising symptom profiles that delay or alter diagnostic recognition, particularly for ADHD (Hinshaw et al., 2022). Treatment duration may reflect the degree of diagnostic refinement achieved over time, consistent with clinical staging models proposing that diagnostic clarity increases as illness trajectories unfold longitudinally (McGorry et al., 2007). Clinician profession may capture systematic differences in diagnostic training, interviewing style, and threshold‐setting, which are established sources of diagnostic disagreement in psychiatry (Di Forti et al., 2025). Internalising and externalising symptom profiles often precede or accompany diagnostic reclassification, particularly as externalising presentations evolve into mood or anxiety disorders during adolescence (Franke et al., 2018; Nikstat & Riemann, 2020).

Moreover, we anticipate that baseline factors, such as patient and clinician characteristics, duration of CAMHS care, and clinician‐rated illness severity, will help predict whether a diagnosis remains stable or changes. Additionally, the type of service transitioned into and the clinician's characteristics at follow‐up are expected to influence the likelihood of diagnostic reclassification.

## METHODS

### Study design and participants

The MILESTONE study was designed to investigate service utilisation, mental health, and additional outcomes during a 2‐year monitoring period (2017–2019) among a group of 1004 young individuals aged 17–19 who have reached the upper age limit of their CAMHS across eight European countries (Belgium, Croatia, France, Germany, Ireland, Italy, the Netherlands, and the UK). Previous studies have described their study design and recruitment procedures in detail (for a thorough review of the research protocol, please refer to Singh et al., 2017). We selected patients from the entire sample with available diagnoses and clinical characteristics at least at two‐time points (*N* = 770) (Supporting Information S1: Figure S1). We chose the last observation carried forward (LOCF) method to handle missing clinical diagnoses because it allows for the preservation of longitudinal continuity in diagnostic data, which was essential for our focus on diagnostic stability. LOCF is a conservative approach that avoids introducing artificial diagnostic changes due to missing values and has been commonly used in psychiatric research for this purpose (Hamer & Simpson, 2009). Participants were considered eligible based on the following criteria: (i) they were users of CAMHS and either approaching the upper age limit of 18 years (with a maximum of 1 year remaining) or had recently turned 18 (mean age = 17.68; SD = 0.29) while still receiving care from CAMHS during the study; (ii) they possessed a documented psychiatric diagnosis or consistently attended CAMHS sessions; (iii) they had an IQ above 70 or demonstrated no signs of intellectual disability, as evaluated by clinical assessments or prior diagnoses; and (iv) they demonstrated sufficient cognitive and emotional functioning to engage meaningfully with the clinical process and successfully complete all required assessments, including clinician‐ and self‐rated measures administered.

### Measures, procedures, and variables

Patients took part in four assessments over a 24‐month follow‐up period: 3 months before transitioning from CAMHS to AHMS (T1), 9 months after T1 (T2), 15 months after T1 (T3), and 24 months after T1 (T4). At T1, all patients received CAMHS treatment. Clinicians and care providers were contacted and given distinct information sheets and consent forms, because they were enroled as secondary participants, to provide information. They qualified for participation if they were the primary clinicians for the young person at CAMHS (or AMHS or another relevant service provider, if a referral has occurred) and provide valid written informed consent. Following informed consent, participants and parents took part in a baseline assessment (T1) in CAMHS approximately 6 months before reaching the upper age limit. Trained researchers collected sociodemographic information and evaluated care needs through interviews held at CAMHS, in participants' homes, or via telephone, depending on their preferences. The sociodemographic data were obtained from a variety of sources, including clinicians, parents, medical records, and online questionnaires accessed through HealthTracker™ (https://www.healthtracker.co.uk). The assessment battery included several structured rating scales (for more information see Supporting Information S1: Table S1). Clinicians of the patients made the primary current diagnosis in a first moment, which was confirmed by research assistant using the SCID‐CV interview at the enrolling. The same procedure was used at each follow‐up. Comorbid diagnoses where also detected, using the SCID‐CV, following the same method. Primary and comorbid diagnoses were assigned independently at each assessment. Across the follow‐up period, no comorbid diagnosis recorded at a given assessment was subsequently reclassified as the primary diagnosis at a later time‐point; diagnostic changes captured by the stability outcome therefore reflected transitions towards categories not previously present in the patient’s diagnostic profile, rather than a reordering of pre‐existing comorbidities. We used diagnoses made by the primary clinicias of the patients, who decided to participate in the study. In our database, adolescents have been categorised into 14 distinct diagnostic groups based on their primary diagnoses at baseline: ADHD, Anxiety, Autism Spectrum Disorder (ASD), Bipolar Disorder, Conduct Disorders, Depression, Eating Disorders (ED), obsessive compulsive disorder (OCD), Personality Disorders, Schizophrenia Spectrum Disorders (SSD), Somatic Disorders, Substance‐Use Disorders (SUD), Trauma‐stress disorders, and None. While this detailed categorisation captured the broad spectrum of mental health diagnoses in our cohort, the large number of diagnostic groups posed significant challenges for meaningful statistical analyses and interpretation. To address this, we opted to reduce the number of diagnostic groups by merging those with overlapping clinical features (Stein et al., 2022), similar patterns of progression, and comparable outcomes. This approach enabled us to preserve the clinical relevance of the diagnostic categories while improving the feasibility and robustness of our analyses. Specifically, we consolidated the 14 diagnostic groups into 10 broader categories as follows:

1. ADHD (unchanged);
2. Anxiety and Trauma‐stress disorders—both characterised by heightened emotional distress, dysregulated stress responses, and overlapping treatment approaches;
3. ASD (unchanged);
4. Depression (unchanged);
5. Bipolar Disorder (unchanged);
6. ED (unchanged);
7. OCD (unchanged);
8. Personality and Conduct Disorders—combined due to their shared externalising behaviours, interpersonal difficulties, and chronic trajectories;
9. SSD (unchanged);
10. SUD (unchanged).

This reduction allowed us to retain the clinical and diagnostic diversity of the sample while facilitating more sophisticated statistical analyses. The revised structure provided a clearer framework for examining longitudinal changes in diagnoses and yielded more interpretable findings regarding diagnostic transitions and outcomes over the 2‐year follow‐up period. We did not include the ‘None’ category in the analysis, as we aimed to focus exclusively on diagnostic stability. Including remissions would have introduced a distinct clinical process—related to treatment response or the natural course of illness—rather than changes or confirmations in diagnostic classification, which were the specific focus of this study.

### Statistical analysis

Data were described using mean and standard deviation (SD) or median and interquartile range for quantitative variables, while qualitative variables were presented as counts and percentages. For the analyses, a dichotomous outcome is constructed. This new variable takes the value 1 if there is a change in diagnosis at each time point compared to the previous time point, and 0 otherwise. We utilised a Sankey plot to illustrate diagnosis transitions across all four time points, showing individuals’ movements between different diagnoses at each point. We included the following predictors in the model based on theoretical and clinical assumptions about their potential association with diagnostic change: patient sex, treatment duration, clinician profession, Achenbach System of Empirically Based Assessment (ASEBA) internalizing and externalizing scores, HoNOSCA, Specific Level of Functioning (SLOF), and Clinical Global Impression Scale (CGI). Sex was considered to explore potential diagnostic biases or differential symptom expression across genders. Longer treatment duration was hypothesized to increase diagnostic refinement over time. The clinician's profession was included to account for differences in training and diagnostic approach. ASEBA scores were used to capture symptom patterns that might prompt diagnostic revision. HoNOSCA and SLOF scores were included as indicators of clinical complexity and functional status, which may influence diagnostic reassessment. Lastly, CGI was used to reflect global clinical severity, potentially affecting the likelihood of a diagnostic change. To ensure model convergence, numerical variables were standardised, and several optimiser was used such as ‘Nlminbwrap’, ‘bobyqa’, etc. (Bates, 2014), but none achieved satisfactory convergence. The model was re‐estimated using the ‘glmmTMB’ (Brooks et al., 2017) package, which offers increased flexibility and stability for fitting generalized mixed models, especially in scenarios with complex model structures. We opted to include a random effect only for the patient and not for the country, as the variability associated with the country was found to be minimal. Analyses were conducted using R software version 4.3.2 with a significance threshold of 0.05.

## RESULTS

### Sociodemographic and clinical characteristics of participants

Table 1 presents the sociodemographic characteristics of 770 adolescent patients and clinicians from nine European centres. The sample had a mean age of 17.5 years (SD = 0.6), with over 78.0% identified as Caucasian and 61.6% as female. The clinical scales reveal a mean internalising score on the Adult Behaviour Checklist–Child Behaviour Checklist of 17.6 (SD = 10.5) and a mean score for externalising problems of 10.6 (SD = 9.5). The average score on the HoNOSCA was 13.4 (SD = 7.7), while the total SLOF scores averaged 187.3 (SD = 20.2). According to Clinician rated severity of psychopathology ratings, 8.7% of patients were classified as ‘not ill’, while 59.9% were categorised as ‘borderline, mildly, or moderately ill’. Additionally, 21.4% were classified as ‘markedly ill or more severe’. At the baseline, the largest group comprises individuals with anxiety and trauma‐related disorders, accounting for 25.1%. This is followed by those diagnosed with depression at 20.9%, ADHD at 15.6%, and ASD at 14.2%. Smaller groups include individuals with ED (8.1%), personality and conduct disorders (8.6%), and obsessive‐compulsive disorder (3.5%). The least represented diagnostic categories are SSD (2.9%), SUD (0.7%), and bipolar disorder (0.4%). Most of the patients had been in the mental health care system for less than 5 years (70.5%). Most of the clinicians were psychiatrists (46.5%), followed by psychotherapists (24.9%) and therapists (i.e., occupational therapists, nurses, family therapist, supporting workers) (6.9%).

**TABLE 1:** Sociodemographic and clinical characteristics of patients and clinicians at baseline (*N* = 770).

Variable	*N* (%) or mean (SD)
Patients	
Gender	
Male	296 (38.4%)
Female	474 (61.6%)
Age	17.5 (0.6)
Ethnicity	
Caucasian	604 (78.4%)
Other ethnic groups	71 (9.3%)
Missing	95 (12.3%)
Country	
Belgium	73 (9.4%)
Croatia	49 (6.4%)
France	83 (10.8%)
Germany	83 (10.8%)
Ireland	39 (5.1%)
Italy	159 (20.6%)
Netherlands	131 (17.0%)
UK London	55 (7.1%)
UK West Mids	98 (12.8%)
ABCL‐CBCL internalising	17.6 (10.5)
ABCL‐CBCL externalising	10.6 (9.5)
HoNOSCA	13.4 (7.7)
SLOF total	187.3 (20.2)
Clinician rated severity of psychopathology (CGI‐S)	
Not at all ill	67 (8.7%)
Borderline/mildly/moderately ill	461 (59.9%)
Markedly ill or more severe	165 (21.4%)
Missing	77 (10.0%)
Diagnosis	
ADHD	120 (15.6%)
Anxiety/trauma	193 (25.1%)
ASD	109 (14.2%)
Depression	161 (20.9%)
Bipolar	3 (0.4%)
ED	62 (8.1%)
OCD	27 (3.5%)
Personality/conduct	66 (8.6%)
SSD	22 (2.9%)
SUD	7 (0.7%)
Average time in treatment (years)	
<5 years	540 (70.2%)
>5 years	192 (24.9%)
Missing	38 (4.9%)
Clinicians	
Profession	
Psychiatrist	358 (46.5%)
Psychotherapist	192 (24.9%)
Therapist	53 (6.9%)
Other	38 (4.9%)

Abbreviations: ABCL, Adult Behaviour Checklist; ADHD, attention‐deficit/hyperactivity disorder; CBCL, Child Behaviour Checklist; CGI‐S, Clinical Global Impression Scale; ED, eating disorders; OCD, obsessive compulsive disorder; SLOF, Specific Level of Functioning; SSD, schizophrenia spectrum disorders; SUD, substance‐use disorders.

### Diagnosis transition over time

Table 2 and 3 and Figure 1, illustrate the changes and stability in mental health diagnoses over time. The data indicate a high level of stability across most diagnostic categories. ADHD (94.2%), Anxiety/Trauma (88.1%), ASD (89.9%), ED (93.5%), and OCD (81.5%) showed that the majority of individuals retained their original diagnosis. In contrast, the Depression group (80.7%) showed moderate stability, with a proportion of individuals transitioning to Anxiety/Trauma or Bipolar diagnoses. Additionally, disorders like SSD (100.0%) and SUD (85.7%) exhibited nearly perfect diagnostic consistency over time, as almost all individuals maintained their initial diagnosis. Diagnostic stability rates varied across the nine participating countries, ranging from 79.5% (Ireland) to 100% (France) (*χ* ^2^ = 32.55, *p* < 0.001, Cramér’s *V* = 0.21, indicating a small‐to‐moderate effect). This variability may partly reflect differences in referral pathways, diagnostic practices, or sample composition across sites, and should be interpreted with caution given the smaller sample sizes in some countries. Overall, the findings suggest that most mental health conditions tend to remain stable over time, with relatively few transitions between diagnostic categories.

**TABLE 2:** Diagnosis transitions from the first time point to the last time point (*N* = 770).

*N* = 770	ADHD (T4, *N* = 120)	Anxiety/trauma (T4, *N* = 184)	ASD (T4, *N* = 106)	Depression (T4, *N* = 151)	Bipolar (T4, *N* = 14)	ED (T4, *N* = 60)	OCD (T4, *N* = 24)	Personality/conduct (T4, *N* = 81)	SSD (T4, *N* = 24)	SUD (T4, *N* = 6)	Stable (%)
ADHD (T1, *N* = 120)	113	‐	2	2	1	‐	‐	1	1	‐	94.2
Anxiety/trauma (T1, *N* = 193)	2	170	1	13	‐	1	1	5	‐	‐	88.1
ASD (T1, *N* = 109)	4	3	98	2	‐	‐	‐	1	1	‐	89.9
Depression (T1, *N* = 161)	1	7	2	130	11	1	‐	9	‐	‐	80.7
Bipolar (T1, *N* = 3)	‐	‐	‐	‐	2	‐	‐	1	‐	‐	66.7
ED (T1, *N* = 62)	‐	‐	1	1	‐	58	‐	2	‐	‐	93.5
OCD (T1, *N* = 27)	‐	1	1	2	‐	‐	22	1	‐	‐	81.5
Personality/conduct (T1, *N* = 66)	‐	2	1	1	‐	‐	1	61	‐	‐	92.4
SSD (T1, *N* = 22)	‐	‐	‐	‐	‐	‐	‐	‐	22	‐	100.0
SUD (T1, *N* = 7)	‐	1	‐	‐	‐	‐	‐	‐	‐	6	85.7

Abbreviations: ADHD, attention‐deficit/hyperactivity disorder; ED, eating disorders; OCD, obsessive compulsive disorder; SSD, schizophrenia spectrum disorders; SUD, substance‐use disorders.

**TABLE 3:** Number of subjects changing diagnosis over time.

Diagnosis (T1)	T1 → T2	T2 → T3	T3 → T4
ADHD (*N* = 120)	3 (2.5%)	4 (3.3%)	3 (2.5%)
Anxiety/trauma (*N* = 193)	13 (6.7%)	12 (6.2%)	7 (3.6%)
ASD (*N* = 109)	9 (8.3%)	2 (1.8%)	3 (2.7%)
Depression (*N* = 161)	17 (10.6%)	13 (8.1%)	8 (5.0%)
Bipolar (*N* = 3)	1 (3.3%)	0 (0.0%)	1 (3.3%)
ED (*N* = 62)	3 (4.8%)	0 (0.0%)	2 (3.2%)
OCD (*N* = 27)	4 (14.8%)	1 (3.7%)	1 (3.7%)
Personality/conduct (*N* = 66)	4 (6.1%)	1 (1.5%)	2 (3.0%)
SSD (*N* = 22)	1 (4.5%)	1 (4.5%)	1 (4.5%)
SUD (*N* = 7)	1 (14.3%)	0 (0.0%)	0 (0.0%)

Abbreviations: ADHD, attention‐deficit/hyperactivity disorder; ED, eating disorders; OCD, obsessive compulsive disorder; SSD, schizophrenia spectrum disorders; SUD, substance‐use disorders.

**FIGURE 1:**
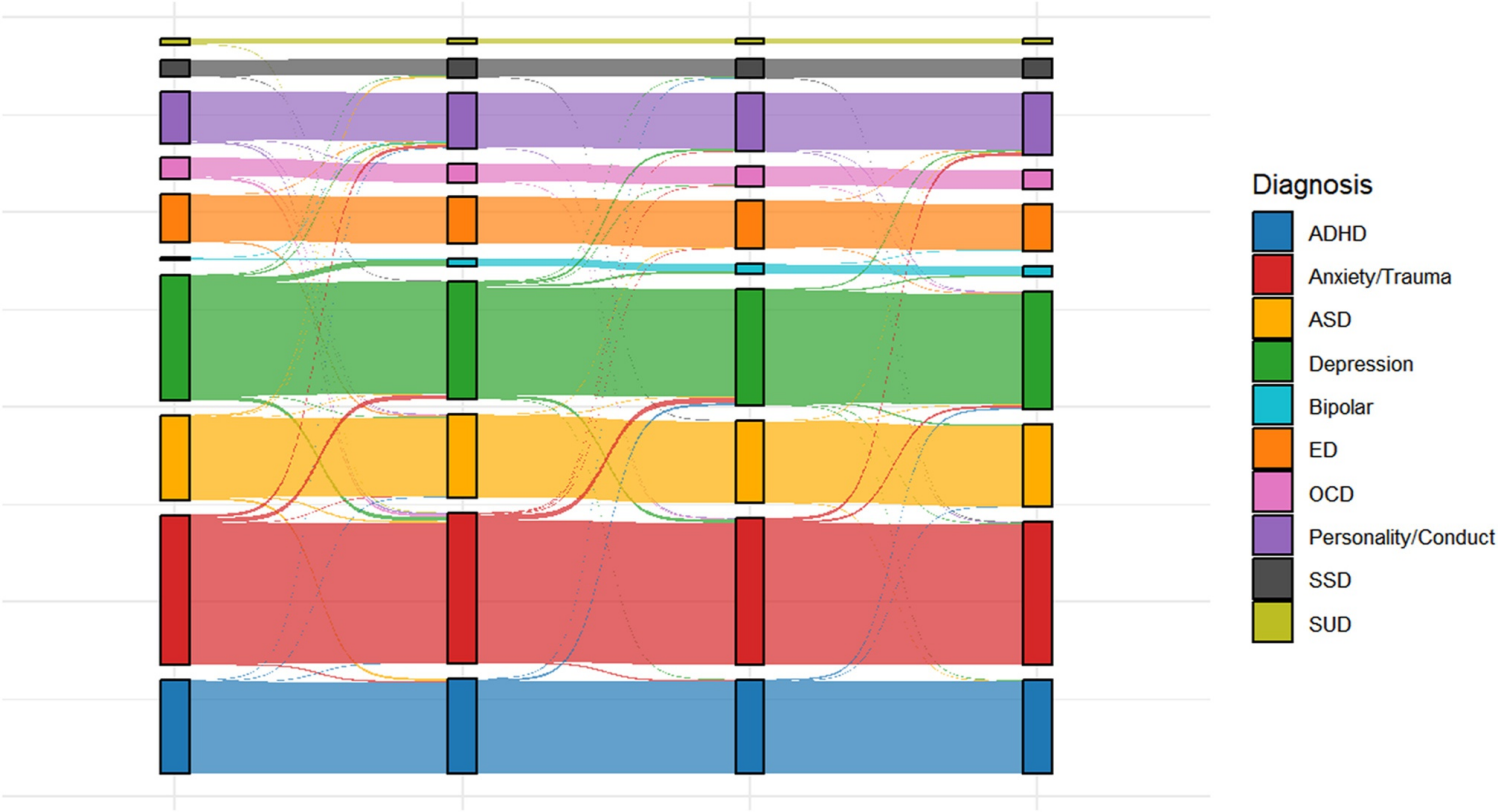
Diagnostic variability of mental disorders in adolescence: A longitudinal study across transition boundary age.

### Predictors of diagnostic change

Table 4 summarises the results of a multilevel logistic regression model that evaluates the relationship between various variables and the outcome of interest which describes the change of diagnoses during the 2‐years follow‐up. The ASEBA Externalising total ratings demonstrate a statistically significant association with the outcome, with an odds ratio of 3.80 (95% CI [1.183–12.201], *p* = 0.025). This suggests that higher externalising scores are significantly linked to an increased likelihood of the outcome. Additionally, the odds ratio related to the profession of psychiatrist is statistically significant (*p* = 0.042), indicating a reduced risk in diagnoses changing. Other variables, including gender, ASEBA internalising scores, SLOF, and CGI total ratings, did not show statistically significant associations. It is important to note that all numerical variables were standardised, meaning the reported risk ratios represent the effect of a one standard deviation change in each respective variable.

**TABLE 4:** Results of multilevel logistic regression model.

Variable	OR^a^	95% CI	*p*‐value
Gender *P* (=male)	0.06	0.001–3.048	0.161
Length service use			
<5 years	0.78	0.082–7.336	0.824
>5 years	‐	‐	‐
Profession			
Psychiatrist	0.01	0.001–0.847	**0.042**
Psychotherapist	0.07	0.001–2.926	0.159
Therapist	0.29	0.003–9.107	0.585
Other	‐	‐	‐
ASEBA internalising	0.61	0.168–2.239	0.459
ASEBA externalising	3.80	1.183–12.201	**0.025**
HONOSCA total	0.45	0.111–1.822	0.265
SLOF total	0.72	0.198–2.626	0.620
CGI	1.97	0.678–5.743	0.212

*Note*: The *p*‐values reported in bold are significant at 0.05 level.

Abbreviations: ASEBA, Achenbach System of Empirically Based Assessment; CGI, Clinical Global Impression Scale; SLOF, Specific Level of Functioning.

^a^ Dependent variable refers to change of diagnoses over time, where 0 = no change and 1 = change.

## DISCUSSION

Our findings should be interpreted against the broader literature on homotypic and heterotypic continuity of psychiatric diagnoses. Homotypic continuity refers to the persistence of the same diagnosis over time, while heterotypic continuity describes a shift from one diagnostic category to another, which some authors argue is inconsistent with viewing psychiatric disorders as discrete, fixed entities (Lahey et al., 2014). Evidence on the relative prevalence of these two patterns is mixed. Some studies, consistent with our findings, report predominant diagnostic stability over time, particularly for certain conditions (Copeland et al., 2013). Others report the opposite pattern: Lahey et al. (2014), in a population‐representative adult sample, found heterotypic continuity to be nearly as pervasive as homotypic continuity, closely tracking the cross‐sectional correlational structure among diagnoses. Similarly, Krantz et al. (2025), in a nationwide Danish cohort of children and adolescents followed for up to 10 years, found diagnostic shifts to be common in CAMHS populations, especially among those whose first psychiatric contact occurred in adolescence, and reported lower diagnostic stability among girls. Several methodological differences may explain the divergence between these findings and ours: our study focussed on primary diagnoses only, consolidated into 10 broader diagnostic groups rather than individual DSM/ICD categories, was conducted in a clinical, treatment‐seeking sample rather than a population‐representative or register‐based cohort, and covered a comparatively shorter follow‐up window (2 years) at four closely spaced assessments rather than long‐term register‐based trajectories. These design choices likely favour the detection of stability over gradual heterotypic drift, and should be considered when generalising our findings.

### Diagnosis transition over time

Viewed through this framework, our findings indicate that homotypic continuity was the dominant pattern across most diagnostic categories. Disorders such as ADHD, Anxiety/Trauma, ASD, ED and OCD remained largely unchanged, with most individuals retaining their initial diagnosis; SSD and SUD showed near‐perfect diagnostic consistency. This pattern of high homotypic continuity is consistent with prior clinical follow‐up studies: Van Meter et al. (2024) followed 431 children with ADHD over 8 years and found that over half retained the diagnosis; similarly, AN was found to be highly stable over 12 years (Fichter & Quadflieg, 2007), and ASD stability was shown to increase with age (Landa et al., 2022). Where diagnostic change did occur, however, it was not random: the most common shifts in our sample occurred between Anxiety/Trauma, Depression and Bipolar Disorder—that is, largely within the internalising domain, consistent with shared symptomatology, overlapping neurobiological circuits, and the role of chronic stress (Du et al., 2017; Martin et al., 2009). This clustering of heterotypic transitions within a broader psychopathological domain mirrors the dimensional pattern described by Lahey et al. (2014), who found that heterotypic continuity, while widespread, closely followed the cross‐sectional correlational structure among diagnoses—that is, disorders were more likely to predict later diagnoses within the same higher‐order domain (e.g., internalising) than across domains. Less frequent transitions between mood and personality disorders in our sample likely reflect the well‐documented diagnostic overlap and developmental variability of personality pathology in adolescence (Chanen & McCutcheon, 2013; Skodol et al., 2010). Overall, our results suggest a picture more consistent with predominant homotypic continuity than with the pervasive heterotypic drift reported by Krantz et al. (2025) in a nationwide Danish cohort of children and adolescents followed over 10 years, where diagnostic shifts were common, particularly among adolescent‐onset cases and among girls. Several factors may account for this divergence. First, our study tracked primary diagnoses consolidated into 10 broad diagnostic groups rather than individual DSM/ICD categories, which likely absorbs some of the within‐domain diagnostic fluctuation that a more granular classification would capture as ‘change’. Second, our sample was a clinical, treatment‐seeking CAMHS‐to‐AMHS transition cohort assessed over a comparatively short 2‐year window across four closely spaced assessments, whereas Krantz et al. (2025) used register‐based diagnoses over a 10‐year horizon, which affords far greater opportunity to observe diagnostic evolution. Third, differences in age range and case‐mix between a population/register‐based cohort and a clinical sample already engaged in specialist mental health services may also contribute to differing baseline diagnostic complexity and stability.

### Predictors of diagnostic change

The predictors associated with diagnostic change in our model are also interpretable within this framework. Higher ASEBA Externalising scores were associated with increased odds of diagnostic change (OR = 2.90, *p* = 0.045), consistent with the heterotypic transitions we observed from externalising to internalising presentations (e.g., ADHD reinterpreted as a depressive disorder; Nikstat & Riemann, 2020) and with Krantz et al.’s (2025) observation that diagnostic instability is concentrated in specific subgroups rather than distributed evenly across the population. Being assessed by a psychiatrist was associated with reduced odds of diagnostic change (*p* = 0.042), suggesting that clinician‐level factors, such as training and diagnostic thresholds—may also moderate whether a case is captured as homotypic or heterotypic continuity, in line with evidence on clinician‐related sources of diagnostic variability (Di Forti et al., 2025). Notably, patient sex was not a significant predictor of diagnostic change in our model, which contrasts with Krantz et al.’s (2025) finding of lower diagnostic stability among girls; this discrepancy may again reflect differences in follow‐up length, diagnostic granularity, and sample composition between the two studies, and warrants further investigation in larger, longer‐term clinical cohorts.

## CONCLUSIONS AND LIMITATIONS

Adolescence is a phase in which the brain develops strongly, emotions grow and identity is formed, making it a complex phase for the diagnosis of mental illness. The expression of symptoms can vary depending on context and developmental stage, making accurate diagnosis difficult. Comprehensive assessments are essential during this period to distinguish between different disorders and avoid misdiagnosis. A key strength of this study is the use of systematic diagnostic assessments conducted in eight European countries. However, the fact that all participants were Caucasian limits the generalisability of the results. Other factors, such as family and socioeconomic status, may also influence diagnostic stability, particularly through differences in access to care. Additionally, our predictor battery, while spanning patient, clinical, and provider‐level domains, remains relatively limited: it does not include environmental or contextual factors (e.g., family functioning, school or peer environment), treatment type or intensity, or the burden of comorbid diagnoses, all of which may independently affect diagnostic stability. Future studies should consider a broader predictor set to more comprehensively capture the determinants of diagnostic change. Possible selection biases must be considered, including the possible exclusion of individuals with more severe disease or the overrepresentation of stable cases, which could inflate stability rates. The ‘psychiatrist’ category did not distinguish between child/adolescent and general psychiatrists. Specialist training pathways in this area differ structurally across the participating countries. This heterogeneity should be considered when interpreting findings related to clinician profession. Another limitation is that the study relies on ASEBA totals without examining how changes in symptoms over time relate to changes in diagnosis. While some diagnostic changes may reflect improved clinical understanding, the design does not allow us to assess whether they occurred with evolving or stable symptom profiles. As adolescents mature, they may overcome symptoms, especially with effective treatment or improved environmental conditions In the multilevel model, the random effect for country was minimal, indicating consistent associations between predictors and diagnostic change across sites. However, diagnostic stability rates varied significantly by country (79.5%–100%). This suggests that country‐level factors, such as diagnostic conventions and service organization, may influence the likelihood of diagnostic change beyond individual‐level characteristics. These findings also have practical implications for CAMHS‐to‐AMHS transition services. The predominant diagnostic stability observed suggests that existing diagnostic information could often be transferred rather than routinely reassessed, whereas young people with internalising diagnoses, where diagnostic shifts were more common, may benefit from more structured clinical review at transition. The association between psychiatrist‐led assessment and greater stability also points to the value of ensuring specialist psychiatric input during this process. Early, accurate diagnosis remains essential to guide intervention and improve long‐term mental health outcomes.

## AUTHOR CONTRIBUTIONS

**Marta Magno**: Conceptualization; validation; writing – original draft; writing – review and editing; visualization. **Donato Martella**: Methodology; software; validation; formal analysis; data curation. **Silvia Leone**: Conceptualization; validation; writing – review and editing; writing – original draft; visualization. **Adriana Pastore**: Conceptualization; writing – original draft. **Cosmo Strozza**: Methodology; data curation; writing – original draft. **Elisa Caselani**: Conceptualization; validation; writing ‐ review and editing. **Samuele Cortese**: Validation; writing – review and editing; supervision. **Miriam D’Addazio**: Writing – review and editing. **Gwen Dieleman**: Writing – review and editing; supervision. **Tomislav Franic**: Writing – review and editing; supervision. **Suzanne Gerritsen**: Writing – review and editing; supervision. **Athanasios Maras**: Writing – review and editing; supervision. **Fiona McNicholas**: Writing – review and editing; supervision. **Diane Purper–Ouakil**: Writing – review and editing; supervision. **Paramala Santosh**: Writing ‐ review and editing; supervision. **Ulrike M. E. Schulze**: Writing – review and editing; supervision. **Cathy Street**: Writing – review and editing; supervision. **Swaran Singh**: Writing – review and editing; supervision; project administration; funding acquisition. **Sabine Tremmery**: Writing – review and editing; supervision. **Helena Tuomainen**: Writing – review and editing; supervision. **Larissa S. van Bodegom**: Writing – review and editing; supervision. **Dieter Wolke**: Writing – review and editing; supervision. **Giovanni de Girolamo**: Writing – review and editing; supervision; project administration; conceptualization; writing – original draft.

## CONFLICT OF INTEREST STATEMENT

S.C. has declared reimbursement for travel and accommodation expenses from the Association for Child and Adolescent Central Health (ACAMH) in relation to lectures delivered for ACAMH, the Canadian AADHD Alliance Resource, the British Association of Psychopharmacology, Healthcare Convention and CCM Group team for educational activity on ADHD, and has received honoraria from Medice. The remaining authors have declared that they have no competing or potential conflicts of interest.

## ETHICAL CONSIDERATIONS

Informed consent and parental consent have been appropriately obtained. The project was approved by the local Ethics Committee of IRCCS Fatebenefratelli (Brescia) Italy on 4th December 2014 (opinion number 70/2014). The authors affirm that all procedures involved in this work adhere to the ethical standards set by the appropriate national and institutional committees on human experimentation, in accordance with the Helsinki Declaration of 1975, as amended in 2008.

## Supporting information

Supporting Information S1

## Data Availability

The data that support the findings of this study are available from the corresponding author upon reasonable request.
