# Supplementary material for: Diagnostic variability of mental disorders in adolescence: A longitudinal study across transition boundary age
Source: JCPP Adv. 2026 Oct 9:e70174. Online ahead of print. doi: 10.1002/jcv2.70174 (PMC13653041; doi:10.1002/jcv2.70174)
Supplement: Supplementary file 1 [file JCV2-9999-e70174-s001.docx]

**DIAGNOSTIC VARIABILITY OF MENTAL DISORDERS IN ADOLESCENCE:**

**A LONGITUDINAL STUDY ACROSS TRANSITION BOUNDARY AGE**

**Supporting Information**

**Table S1.**

**LIST OF STANDARDISED TOOLS USED**

| **TOOLS** | **DESCRIPTION** | **RANGE** | **ADMINISTERED TO** |
| --- | --- | --- | --- |
| Achebanch System of Empirically Based Assessment (ASEBA) | The Achenbach System of Empirically-Based  Assessment [ASEBA] have been used extensively in different contexts to evaluate emotional and behavioural problems in the last 6 months. | Higher scores indicate more emotional/behavioural problems | Patients and parents |
| Clinical Global Impression – Severity scale (CGI-S) | Instrument to measure the severity of the disorder | 1-7 | Clinicians |
| Health of the Nation Outcome Scale for Children and Adolescents (HoNOSCA) | The Health of the Nation Outcome Scale for Children and Adolescents [HoNOSCA] measures general health and social functioning in child and adolescent attending mental health services | 0-52 | Patients, parents and clinicians |
| World Health Organization Quality of Life Brief Inventory (WHOQOL-BREF) | Self-administered questionnaire to assess subjective health and well-being over the previous two weeks. It covers four domains: physical health, psychological health, social relationships, and environment. | 0-100 | Patients |
| Specific Levels of Functioning (SLOF) | To assess psychosocial functioning in six subscales: physical functioning, self-care skills, interpersonal relationships, social acceptance, community life activities, and work skills. | 43-125 | Parents |
| Life events | Instrument developed specifically for this project to assess life events | 0-13 | Patients |
| Retrospective Bullying and Friendship Interview Schedule (adapted version) | Instrument used to assess the patient experiences with bullying in different settings | NA | Patients |

**FIGURE S1
Flowchart of the study**
